# Chronic rhinosinusitis in adolescence is a rare but bothersome condition - data from a Swedish population based cohort

**DOI:** 10.1186/2045-7022-5-S4-P27

**Published:** 2015-06-26

**Authors:** Marit Westman, Par Stjarne, Anna Bergstrom, Inger Kull, Elina Toskala, Lars Olaf Cardell, Magnus Wickman, Mats Holmstrom

**Affiliations:** 1 Karolinska Institutet, CLINTEC, Stockholm, Sweden; 2 Karolinska Institutet, Institute of Environmental Medicine, Stockholm, Sweden; 3 Temple University, Dept. of Otolaryngology-Head and Neck Surgery, Philadelphia, USA

## Background

Symptoms of chronic rhinosinusitis (CRS) is common, around 10% among adults, and has shown to be associated with reduced quality of life. No study has focused on adolescents specifically. Therefore, we wanted to estimate the prevalence of CRS in adolescence and evaluate the burden of symptoms.

## Method

We used 3112 16-year-olds from a Swedish birth cohort called BAMSE. The adolescents who fulfilled the criteria of CRS according to the European Position Paper on Rhinosinusitis (EPOS) in a questionnaire, was contacted by telephone to verify the diagnosis. Those with ongoing symptoms were invited for a clinical follow up with nasal endoscopy, olfactory threshold test (Sniffin’ sticks) and the disease specific quality-of-life (QoL) questionnaire SNOT-22 (Sino Nasal Outcome Test 22). QoL was compared to those without CRS with the generic EQ-5D VAS.

## Results

Among the 3112 16-year-olds 43.5% reported symptoms from the upper airways during the last 12 months, but only 1.5% (n=48) reported symptoms of CRS. 27 of the 48 adolescents still had ongoing symptoms of CRS at the telephone interview. At clinical examination of 23 of the 27 adolescents, 22 still fulfilled the criteria of CRS among whom 9 had endoscopic signs of CRS, corresponding to a prevalence of 0.3%. The 22 adolescents with symptoms of CRS more often had allergic rhinitis symptoms (57.1% vs 28.1%, p=0.003) asthma (25.0% vs 11.0%, p=0.047) and cough ≥ 3 months (13.6% vs 3.4%, p=0.008) compared to those without symptoms of CRS. The median EQ-5D VAS score was lower compared to the rest of the population (M=80 vs M=90, p=0.024). The mean SNOT-22 value was 38.2 among the 22 adolescents with symptoms of CRS and 44.2 among the 9 adolescents with endoscopic signs of CRS. The corresponding mean values for the olfactory threshold were 6.08 and 6.33, respectively.

## Conclusion

At adolescence CRS exists even though the prevalence is low, between 0.3% and 1.5%. Those affected have a significantly reduced quality of life.

